# Supplementary material for: Cnidom in Ceriantharia (Cnidaria, Anthozoa): new findings in the composition and micrometric variations of cnidocysts
Source: PeerJ. 2023 Jun 21;11:e15549. doi: 10.7717/peerj.15549 (PMC10290448; doi:10.7717/peerj.15549)
Supplement: Supplemental Information 2 — N: number of samples. [file peerj-11-15549-s002.pdf]

Table S1:
Specimens collected and used in this study.
N: number of specimens.

**Species**			
Country	Locality	Coordinates	Voucher code
Ceriantheomorphe brasiliensis			
Brazil	Camburi Beach, Vitória, Espírito Santo	20°16'39"S, 40°16'29"W	UFRJ Biologia 0293/0337
	Arraial do Cabo, Rio de Janeiro	23°0'4"S, 42°0'29"W	MZSP 8470
	Cagarras Archipelago, Rio de Janeiro, Rio de Janeiro	23°1'55"S, 43°11'58"W	MZSP 8473
	Guanabara Bay, Rio de Janeiro, Rio de Janeiro	22°49'6"S, 43°8'45"W	MNRJ 200
	Araçá Beach, São Sebastião, São Paulo	23°48'58"S, 45°24'24"W	MZSP 8472
	Pitangueiras Beach, São Sebastião, São Paulo.	23°49'22"S 45°24'36"W	LEDAFCZOO 02
	Praia de Canasvieiras, Florianópolis, Santa Catarina	27°25'31"S, 48°27'02"W	LEDAFCZOO 01
Uruguay	Montevideo	34°50'S 55°40'W	LEDAFCZOO 03
	La Paloma, Rocha	34°42'3"S, 54°0.5'W	UFRJ-Biologia 2-464 A
Cerianthus sp.			
Uruguay	Punta del Este, Maldonado	34°42'3"S, 54°0.5'W	LEDAFCZOO 04-08
Argentina	Mar del Plata, Buenos Aires	38°06'15"S 57°32'07"W	LEDAFCZOO 09-10

Voucher collection acronyms: UFRJ (Federal University of Río de Janeiro), MZSP (Museum of Zoology of the University of São Paulo), LEDAFCZOO (LEDA Laboratory collection).
