# Supplementary material for: Cnidom in Ceriantharia (Cnidaria, Anthozoa): new findings in the composition and micrometric variations of cnidocysts
Source: PeerJ. 2023 Jun 21;11:e15549. doi: 10.7717/peerj.15549 (PMC10290448; doi:10.7717/peerj.15549)
Supplement: Supplemental Information 3 — P-values of the t test for GLM coefficients (ß1) for each cnidocyst type. Underlined P-values significant at α = 0.05. [file peerj-11-15549-s003.pdf]

Table S2a:
***Ceriantheomorphe brasiliensis*. Comparison of cnidocyst lengths between individuals in the actinopharynx, column and metamesenteries.**

P-values of the t test for GLM coefficients (β_1) for each cnidocyst type. Underlined *P*-values significant at α=0.05.

	**Actinopharynx**	**Column**			**Metamesenteries**	
**Specimen**	**atrich I**	**atrich II**	**microbasic b-mastigophore I**	**ptychocyst**	**microbasic b-mastigophore I**	**microbasic b-mastigophore VI**
1	++	++	0.019	++	++	//
2	//	0.012	++	0.037	//	++
3	<0.001	<0.001	0.005	0.615	//	0.182
4	<0.001	0.904	0.014	<0.001	<0.001	//
5	0.0974	<0.001	<0.001	<0.001	//	//
6	<0.001	0.113	0.393	0.057	//	//
7	<0.001	<0.001	<0.001	<0.001	//	//
8	<0.001	<0.001	<0.001	//	0.013	0.001
9	<0.001	0.111	<0.001	//	0.092	//
10	0.058	<0.001	<0.001	<0.001	0.494	<0.001

Notes:
++: cnidocyst used for comparisons; //: cnidocysts not found in the structure.

Table S2b:
***Ceriantheomorphe brasiliensis*. Comparison of cnidocyst lengths between individuals in marginal tentacles.**

P-values of the t test for GLM coefficients (β_1) for each cnidocyst type. Underlined *P*-values significant at α=0.05.

	**Marginal Tentacles**				
**Specimen**	**microbasic b-mastigophore I**	**microbasic b-mastigophore II**	**microbasic b-mastigophore III**	**microbasic b-mastigophore I**	**microbasic b-mastigophore VI**
1	++	++	//	++	//
2	//	<0.001	//	<0.001	//
3	0.483	<0.001	++	0.001	++
4	//	<0.001	<0.001	//	<0.001
5	<0.001	<0.001	<0.001	0.046	<0.001
6	0.454	<0.001	<0.001	<0.001	<0.001
7	<0.001	<0.001	<0.001	//	<0.001
8	//	<0.001	<0.001	//	<0.001
9	<0.001	<0.001	<0.001	<0.001	<0.001
10	<0.001	<0.001	0.139	<0.001	//

Notes:
++: cnidocyst used for comparisons; //: cnidocysts not found in the structure.

Table S2c:

***Ceriantheomorphe brasiliensis*. Comparison of cnidocyst lengths between individuals in labial tentacles.**

P-values of the t test for GLM coefficients ($ß_1$) for each cnidocyst type. Underlined *P*-values significant at $\alpha=0.05$.

	Labial Tentacles				
Specimen	atrich	microbasic b-mastigophore I	microbasic b-mastigophore II	microbasic b-mastigophore III	microbasic b-mastigophore V
1	++	++	//	++	++
2	<0.001	<0.001	++	<0.001	<0.001
3	//	<0.001	<0.001	0.035	//
4	<0.001	<0.001	<0.001	<0.001	//
5	<0.001	<0.001	<0.001	0.004	//
6	<0.001	<0.001	<0.001	<0.001	<0.001
7	//	0.837	<0.001	0.051	//
8	<0.001	<0.001	<0.001	<0.001	//
9	<0.001	<0.001	//	<0.001	//
10	0.005	<0.001	<0.001	<0.001	<0.001

Notes:

++: cnidocyst used for comparisons; //: cnidocysts not found in the structure.
