# Supplementary material for: Cnidom in Ceriantharia (Cnidaria, Anthozoa): new findings in the composition and micrometric variations of cnidocysts
Source: PeerJ. 2023 Jun 21;11:e15549. doi: 10.7717/peerj.15549 (PMC10290448; doi:10.7717/peerj.15549)
Supplement: Supplemental Information 4 — Number of individuals which present each cnida type over total number of studied specimens. [file peerj-11-15549-s004.pdf]

Table S3:
Representativeness of each cnidocyst type by level in each structure of *Ceriantheomorphe brasiliensis*.
Number of individuals which presented each cnidocyst type over the total number of specimens studied.

Structure/cnidocyst type	Level		
	low	middle	high
**Actinopharynx**			
atrich	9/10	5/10	5/10
microbasic b-mastigophore I	2/10	2/10	4/10
microbasic b-mastigophore II	1/10	0/10	0/10
microbasic b-mastigophore III	3/10	6/10	4/10
**Column**			
atrich*	10/10	10/10	10/10
microbasic b-mastigophore I	0/10	0/10	4/10
microbasic b-mastigophore V	3/10	1/10	2/10
microbasic b-mastigophore VI	1/10	0/10	0/10
holotrich	0/10	0/10	1/10
ptychocyst	4/10	6/10	1/10
**Metamesenteries**			
microbasic b-mastigophore I	4/10	4/10	4/10
microbasic b-mastigophore IV	0/10	2/10	3/10
**Labial tentacles**			
atrich	8/10	3/10	0/10
microbasic b-mastigophore I*	10/10	10/10	9/10
microbasic b-mastigophore II	5/10	8/10	7/10
microbasic b-mastigophore III*	10/10	10/10	9/10
microbasic b-mastigophore V	2/10	2/10	3/10
**Marginal tentacles**			
atrich	7/10	0/10	0/10
microbasic b-mastigophore I	0/10	4/10	6/10
microbasic b-mastigophore II▲	9/10	8/10	8/10
microbasic b-mastigophore III	1/10	6/10	5/10
microbasic b-mastigophore V	5/10	6/10	5/10
microbasic b-mastigophore VI	5/10	7/10	1/10

Notes:

*present in ≥90% of the specimens studied in each and every level/total specimens studied ▲present in ≥70% of the specimens studied in each and every level/total specimens.
