# Supplementary material for: Cnidom in Ceriantharia (Cnidaria, Anthozoa): new findings in the composition and micrometric variations of cnidocysts
Source: PeerJ. 2023 Jun 21;11:e15549. doi: 10.7717/peerj.15549 (PMC10290448; doi:10.7717/peerj.15549)
Supplement: Supplemental Information 5 [file peerj-11-15549-s005.pdf]

Table S4:
***Ceriantheomorphe brasiliensis*. ANOVA for the LMM or GLMM versus the null model for length of different cnidocyst types from the three levels of column and labial tentacles.**

Cnidocyst type (Structure)/Models	*npar*	AIC	logLik	deviance	Chisq	*Df*	Pr(>Chisq)
**Atrich (Column)**							
Atrich length ~ level ♦	4	5290.2	-2641.1	5282.2			
Atrich length ~ level + (1 |Individual)▲*	5	4798.1	-2394.1	4788.1	494.12	1	<0.001
**Microbasic b-mastigophore I (Labial tentacles)**							
Microbasic b-mastigophore I ~ level◊	4	20700	-10346.1	20692			
Microbasic b-mastigophore I ~ leve l+ (1 | Individual)►*	5	18691	-9340.6	18681	2011	1	<0.001
**Microbasic b-mastigophore III (Labial tentacles)**							
Microbasic b-mastigophore III ~ level◊	4	12693	-6342.6	12685			
Microbasic b-mastigophore III ~ level + (1 | Individual)►*	5	11173	-5581.6	18783	1521.9	1	<0.001

Notes:
***best model.** *npar*: number of parameters; AIC: Akaike Information Criterion for the model evaluated as - *(logLik -npar); logLik: log-likelihood for the model. ♦ null LM; ▲ LMM; ◊null GLM; ► GLMM. Underlined *P*-values significant at α=0.05.
