# Supplementary material for: Cnidom in Ceriantharia (Cnidaria, Anthozoa): new findings in the composition and micrometric variations of cnidocysts
Source: PeerJ. 2023 Jun 21;11:e15549. doi: 10.7717/peerj.15549 (PMC10290448; doi:10.7717/peerj.15549)
Supplement: Supplemental Information 6 — Units: μm. Models fitted: LMM for atrichs from the column; GLMM for microbasic bmastigophores I and III from the labial tentacles. [file peerj-11-15549-s006.pdf]

Table S5:

***Ceriantheomorphe brasiliensis.* Standard deviation (SD) and variance of the random effects of the models fitted for length of cnidocysts.**

Units: µm. Models fitted: LMM for atrichs from the column; GLMM for microbasic b-mastigophores I and III from the labial tentacles.

	SD	variance
**Atrich (Column)**		
Individual	3.974	15.79
Residual	3.922	15.38
**Microbasic b-mastigophore I (Labial tentacles)**		
Individual	1.284	1.648
Residual	0.101	0.01
**Microbasic b-mastigophore III (Labial tentacles)**		
Individual	0.886	0.785
Residual	0.113	0.012
