# Supplementary material for: Cnidom in Ceriantharia (Cnidaria, Anthozoa): new findings in the composition and micrometric variations of cnidocysts
Source: PeerJ. 2023 Jun 21;11:e15549. doi: 10.7717/peerj.15549 (PMC10290448; doi:10.7717/peerj.15549)
Supplement: Supplemental Information 7 — Models fitted: LMM for atrichs from the column; GLMM for microbasic b-mastigophores I and III from the labial tentacles. [file peerj-11-15549-s007.pdf]

Table S6:

***Ceriantheomorphe brasiliensis.* Length of cnidocysts estimated (µm) and confidence intervals (CI) calculated for them in each structure and level by the models.**

Models fitted: LMM for atrichs from the column; GLMM for microbasic b-mastigophores I and III from the labial tentacles.

Cnidocyst type (Structure)/Level		CI	
	Estimated (µm)	lower-95	upper-95
**Atrich (Column)**			
low	53.736	51.229	56.243
middle	50.599	47.439	53.76
high	44.296	41.143	47.45
**Microbasic b-mastigophore I (Labial tentacles)**			
low	44.02	40.392	47.648
middle	42.018	41.648	49.644
high	46.548	42.521	50.575
**Microbasic b-mastigophore III (Labial tentacles)**			
low	25.524	23.247	27.801
middle	23.625	21.11	26.139
high	22.664	20.108	25.219
