# Supplementary material for: Cnidom in Ceriantharia (Cnidaria, Anthozoa): new findings in the composition and micrometric variations of cnidocysts
Source: PeerJ. 2023 Jun 21;11:e15549. doi: 10.7717/peerj.15549 (PMC10290448; doi:10.7717/peerj.15549)
Supplement: Supplemental Information 8 — P-values of the t test for GLM coefficients (ß1) for each cnidocyst type. Underlined P-values significant at α = 0.05. [file peerj-11-15549-s008.pdf]

Table S7a:
***Cerianthus sp*. Comparison of cnidocyst lengths between specimens in the actinopharynx, metamesenteries and marginal tentacles.**
P-values of the t test for GLM coefficients ($ß_1$) for each cnidocyst type. Underlined *P*-values significant at α=0.05.

	**Actinopharynx**	**Metamesenteries**	**Marginal tentacles**		
**Specimen**	**atrich I**	**microbasic b-mastigophore I**	**microbasic b-mastigophore I**	**microbasic b-mastigophore II**	**microbasic b-mastigophore III**
1	++	//	++	++	++
2	0.067	++	//	//	//
3	<0.001	0.126	//	//	//
4	<0.001	0.009	<0.001	<0.001	//
5	<0.001	//	//	//	//
6	<0.001	//	//	<0.001	<0.001
7	//	//	//	//	//

Notes:
++: cnidocyst used for comparisons; //: cnidocysts not found in the structure.

Table S7b:
***Cerianthus sp*. Comparison of cnidocyst lengths between specimens in labial tentacles.**
P-values of the t test for GLM coefficients ($ß_1$) for each cnidocyst type. Underlined *P*-values significant at α=0.05.

	**Labial tentacles**			
**Specimens**	**atrich**	**microbasic b-mastigophore I**	**microbasic b-mastigophore II**	**microbasic b-mastigophore III**
1	++	++	++	++
2	//	<0.001	<0.001	0.12
3	0.701	<0.001	//	<0.001
4	0.225	<0.001	//	<0.001
5	<0.001	<0.001	<0.001	0.827
6	0.15	<0.001	//	0.569
7	<0.001	//	//	//

Notes:
++: cnidocyst used for comparisons; //: cnidocysts not found in the structure.
