# Supplementary material for: Cnidom in Ceriantharia (Cnidaria, Anthozoa): new findings in the composition and micrometric variations of cnidocysts
Source: PeerJ. 2023 Jun 21;11:e15549. doi: 10.7717/peerj.15549 (PMC10290448; doi:10.7717/peerj.15549)
Supplement: Supplemental Information 9 — Number of individuals which present each cnidocyst type over the total number of specimens studied. [file peerj-11-15549-s009.pdf]

Table S8:
Representativeness of each cnidocyst type by level in each structure of *Cerianthus sp*.
Number of individuals which present each cnidocyst type over the total number of specimens studied.

Structure/cnidocyst type	Level		
	low	middle	high
**Actinopharynx**			
atrich *	6/6	6/6	6/6
microbasic b-mastigophore I	5/6	3/6	3/6
microbasic b-mastigophore III	0/6	1/6	1/6
**Column**			
atrich I	5/7	2/7	4/7
atrich II	4/7	3/7	2/7
microbasic b-mastigophore I	0/7	0/7	1/7
ptychocyst	2/7	4/7	3/7
**Metamesenteries**			
microbasic b-mastigophore I *	3/3	3/3	3/3
**Labial Tentacles**			
atrich	6/7	2/7	1/7
microbasic b-mastigophore I▲	6/7	6/7	6/7
microbasic b-mastigophore II	0/7	2/7	2/7
microbasic b-mastigophore III	4/7	6/7	5/7
microbasic b-mastigophore VII	0/7	1/7	1/7
**Marginal Tentacles**			
atrich	3/4	0/4	0/4
microbasic b-mastigophore I	1/4	1/4	2/4
microbasic b-mastigophore II	2/4	1/4	2/4
microbasic b-mastigophore III	1/4	2/4	1/4
microbasic b-mastigophore V	1/4	0/4	0/4
microbasic b-mastigophore VI	2/4	2/4	1/4
microbasic b-mastigophore VII	1/4	1/4	1/4

Notes:

*present in ≥90% of the specimens studiedin each and every level/total specimens; ▲present in ≥70% of the specimens in each and every level/total specimens
