# Supplementary material for: Cnidom in Ceriantharia (Cnidaria, Anthozoa): new findings in the composition and micrometric variations of cnidocysts
Source: PeerJ. 2023 Jun 21;11:e15549. doi: 10.7717/peerj.15549 (PMC10290448; doi:10.7717/peerj.15549)
Supplement: Supplemental Information 10 [file peerj-11-15549-s010.pdf]

Table S9:
***Cerianthus sp*. ANOVA for the GLMM versus the null model for different cnidocyst types from the three levels of the actinopharynx and metamesenteries.**

Cnidocyst type (Structure)/Models	npar	AIC	logLik	deviance	Chisq	Df	Pr(>Chisq)
**Atrich (Actinopharynx)**							
Atrich length ~ level◊	4	3078.2	-1535.1	3070.2			
Atrich length ~ level + (1 |Individual)►*	5	2809	-1399.5	2799	271.22	1	<u><0.001</u>
**Microbasic b-mastigophore I (Metamesenteries)**							
Microbasic b-mastigophore I ~ level ◊*	4	1228.2	-610.1	1220.2			
Microbasic b-mastigophore I ~ level + (1 | Individual)►	5	1229.7	-609.87	1219.7	0.4505	1	0.502

Notes:
***best model.** *npar*: number of parameters; AIC: Akaike Information Criterion for the model evaluated as - *(logLik -npar); logLik: log-likelihood for the model. ◊null GLM; ► GLMM. Underlined values significant at α=0.05.
