# Supplementary material for: Cnidom in Ceriantharia (Cnidaria, Anthozoa): new findings in the composition and micrometric variations of cnidocysts
Source: PeerJ. 2023 Jun 21;11:e15549. doi: 10.7717/peerj.15549 (PMC10290448; doi:10.7717/peerj.15549)
Supplement: Supplemental Information 11 [file peerj-11-15549-s011.pdf]

Table S10:
***Cerianthus sp.* Standard deviation (SD) of random effects from the GLMM for the lengths of atrichs from the actinopharynx.**

	SD	variance	Groups
**Atrich (Actinopharynx)**			
Individual	1.409	1.986	6
Residual	0.016	0.128	
