# Supplementary material for: Cnidom in Ceriantharia (Cnidaria, Anthozoa): new findings in the composition and micrometric variations of cnidocysts
Source: PeerJ. 2023 Jun 21;11:e15549. doi: 10.7717/peerj.15549 (PMC10290448; doi:10.7717/peerj.15549)
Supplement: Supplemental Information 12 — Models fitted: GLMM for atrichs from the actinopharynx; GLM for microbasic b-mastigophores I from the metamesenteries. [file peerj-11-15549-s012.pdf]

Table S11:
***Cerianthus sp.* Length of cnidocysts estimated (µm) and confidence intervals (CI) calculated for them in each structure and level by the models.**
Models fitted: GLMM for atrichs from the actinopharynx; GLM for microbasic b-mastigophores I from the metamesenteries.

Cnidocyst type (Structure)/Level	Estimate (µm)	CI lower-95	CI upper-95
**Atrich (Actinopharynx)**			
low	30.991	27.353	34.628
middle	29.317	24.907	33.726
high	33.806	29.316	38.296
**Microbasic b-mastigophore I (Metamesenteries)**			
low	18.947	18.49	19.42
middle	19.108	17.986	20.246
high	19.316	18.19	20.457
